# Functional foregut anatomy of the blue–green sharpshooter illustrated using a 3D model

**DOI:** 10.1038/s41598-021-85954-4

**Published:** 2021-03-22

**Authors:** Daniel White, Elaine A. Backus, Ian M. Marcus, Sharon L. Walker, M. Caroline Roper

**Affiliations:** 1 Department of Chemical and Environmental Engineering, University of California, Riverside, A220 Bourns Hall, 900 University Ave, Riverside, CA 92521 USA; 2 United States Department of Agriculture, Agricultural Research Service, San Joaquin Valley Agricultural Sciences Center, 9611 South Riverbend Avenue, Parlier, CA 93648-9757 USA; 3 Department of Civil, Architectural, and Environmental Engineering, Drexel University, 3141 Chestnut Street, Philadelphia, PA 19104 USA; 4 Department of Microbiology and Plant Pathology, University of California, Riverside, 900 University Ave, Riverside, CA 92521 USA

**Keywords:** Biophysics, Computational biology and bioinformatics, Microbiology, Physiology, Engineering, Mathematics and computing

## Abstract

Sharpshooter leafhoppers (Hemiptera: Cicadellidae: Cicadellinae) are important vectors of the plant pathogenic bacterium *Xylella fastidiosa* Wells et al. (Xanthomonadales: Xanthomonadaceae). This pathogen causes economically significant diseases in olive, citrus, and grapes on multiple continents. Bacterial acquisition and inoculation mechanisms are linked to *X. fastidiosa* biofilm formation and fluid dynamics in the functional foregut of sharpshooters, which together result in egestion (expulsion) of fluids likely carrying bacteria. One key *X. fastidiosa* vector is the blue–green sharpshooter, *Graphocephala atropunctata* (Signoret, 1854). Herein, a 3D model of the blue–green sharpshooter functional foregut is derived from a meta-analysis of published microscopy images. The model is used to illustrate preexisting and newly defined anatomical terminology that is relevant for investigating fluid dynamics in the functional foregut of sharpshooters. The vivid 3D illustrations herein and supplementary interactive 3D figures are suitable resources for multidisciplinary researchers who may be unfamiliar with insect anatomy. The 3D model can also be used in future fluid dynamic simulations to better understand acquisition, retention, and inoculation of *X. fastidiosa.* Improved understanding of these processes could lead to new targets for preventing diseases caused by *X. fastidiosa*.

## Introduction

Sharpshooter leafhoppers (Hemiptera: Cicadellidae: Cicadellinae) feed on plant tissues using their piercing-sucking mouthparts, termed stylets^1^. They transmit (acquire, retain, and inoculate) the xylem-limited bacterial pathogen *Xylella fastidiosa* Wells et al. (Xanthomonadales: Xanthomonadaceae) during feeding^2^. This invasive, pathogenic bacterium causes $100′s of millions in agricultural losses annually worldwide from plant diseases on multiple continents, including olive quick decline syndrome in South America and Europe, citrus variegated chlorosis in South America, and Pierce’s disease of grape in North America^3^. The state of California alone spends $104 million a year to control *X. fastidiosa*^4^. Of the 2,313 catalogued sharpshooter species worldwide, 35 are reported vectors of *X. fastidiosa*^5^*.* A native California vector, the blue–green sharpshooter, *Graphocephala atropunctata* (Signoret, 1854), has been used in several studies on the mechanism of *X. fastidiosa* transmission^2,6–8^. Thus, this vector species is used as a model for the present study.

When sharpshooters ingest (take up through the stylets and swallow) xylem sap from an infected grapevine using their stylets, *X. fastidiosa* bacteria are acquired as fluid passes through and resides in the functional foregut, eventually to attach and form a biofilm. *Xylella fastidiosa* is later inoculated via egestion when the vector feeds on healthy grapevine xylem^9,10^. Both the acquisition and inoculation mechanisms involve fluid dynamics in the functional foregut (the narrow precibarium leading to the wider cibarium or food pump) during feeding behaviors^2,11,12^. Better understanding of these mechanisms will aid in interpretation of electropenetrography (EPG) data that is being used to select and breed crop varieties that can resist inoculation of the bacterium by its vectors (Backus, *unpublished data*).

Previous studies have modeled fluid flow in the sharpshooter functional foregut. Early attempts at modeling relied on average daily excretion rate as a proxy for ingestion, for both blue–green sharpshooters and glassy-winged sharpshooters^6,13^. Velocities in the precibaria of these sharpshooters were estimated by dividing time-averaged feeding/excretion rates by representative cross-sectional areas of the precibaria. Rapicavoli et al. provided a more precise spatial resolution in the functional foregut by creating a simple two-dimensional model of the precibarium and cibarium of the blue–green sharpshooter^14^. Ranieri et al. created three-dimensional (3D) models of the stylet food canal and precibarium of both spittlebugs and sharpshooters^12^. They modeled the precibarium with finer spatial fidelity than any previously published model by using sequential concentric cylindrical segments of variable diameter. This increased spatial resolution was coupled with increased temporal resolution by using EPG data for estimates of ingestion flowrates. However, their model assumed a continuous distribution of bacterial biofilm.

Herein we propose a 3D model of the blue–green sharpshooter functional foregut that further enhances capabilities for modeling fluid flow through the critical bottleneck for flow, the precibarium. The model is based on a meta-analysis of previously published microscopy images of sharpshooters, incorporating over 30 dimensions of the functional foregut collected from the literature. Our model incorporates complex geometries that provide greater morphological fidelity to the actual functional foregut, especially the precibarium, than previous models. The morphological fidelity of the present model allows for modeling the flowrates close to the precibarial walls in places where the walls curve and bend in ways that are not easily modeled using a cylinder/cone-based 3D model. This will permit assessment of how flow affects surface-attached bacteria at these locations, which will be more relevant for understanding *X. fastidiosa* acquisition, retention, and inoculation.

The anatomical complexity of the sharpshooter functional foregut requires intricate understanding and logical terminology to communicate results effectively^6,7,15–18^. Various terms for the anatomy of the functional foregut, sometimes conflicting, were introduced in previous papers. Therefore, we also seek herein to clarify terms for the anatomical parts and their corresponding regions in the model.

In all hemipterans, the stylet fascicle is the set of mouth parts that are inserted into the plant during feeding. The food canal, formed by grooves in the maxillary stylets, conveys fluid from the plant into the head of the insect, wherein the complete foregut takes over fluid transport. The functional foregut is the segment of the complete foregut that conveys fluid from the proximal opening of the stylet food canal through the precibarium into the cibarium (the sucking pump); the precibarium is also the functional mouth. Fluid then is swallowed through the true mouth (the opening from the cibarium into the pharynx) into the true foregut, consisting of the pharynx then esophagus. Thus, in sharpshooters and other auchenorrhynchan hemipterans, the functional foregut comprises the precibarium and cibarium, while the true foregut comprises the pharynx and esophagus^19,20^. In sharpshooters and other auchenorrhynchan hemipterans, the functional foregut is shaped like a wineglass (i.e., acetabular), whereas in sternorrynchans and heteropterans, it is tubular in shape^19^.

The cibarium functions to both take up fluid from the stylet tips and to swallow fluid past the true mouth into the true foregut (the pharynx and esophagus)^19^. Swallowing of fluid is also termed ingestion^20^. The walls of both the precibarium and cibarium are formed by the apposition of hypopharynx (or hypopharyngeal plate), epipharynx (or epipharyngeal plate), and, in the case of the cibarium, the membranous cibarial diaphragm (the lid of the pump). These walls can be visualized upon dissection of sharpshooter heads, as illustrated previously^7,17,18,21^. Some authors have used the term cibarium chamber for the lumen (cavity) enclosed by these walls^7^. The cibarial walls are composed of the cibarial diaphragm and cibarium bowl^10^. The cibarial diaphragm contains the apodemal groove and pockets of the apodemal groove^15,16^.

The precibarium has complex morphology in a small space. There are four anatomical/functional areas that were originally named in sharpshooters based on SEM of the structures in the lumen (that is, they are lumenal structures)^17^. Progressing distally from the opening of the cibarium to the precibarium, the anatomical structures of the precibarium are the: (1) trough, (2) basin (which houses, in order, the H-[hypopharyngeal] sensilla on that side of the basin, then on the epipharyngeal side, the P [proximal]-sensilla, precibarial valve/flap, and its precibarial pit/ring, (3) D (distal)-sensilla field, and (4) hypopharyngeal extension that inserts into the stylet food canal (HEF)^7,11,17^. The blue–green sharpshooter precibarium contains twenty precibarial chemosensilla, split into six groups that each serve as separate, bilaterally symmetrical gustatory organs. Organ status is based on the arrangement of their neuron cell bodies and the axons/nerve bundles that arise at the precibarium and terminate in the frontal ganglion. The proximal organs each consist of three P-sensilla: one set on the left side of the basin, the other set on the right^18^. The ten D-sensilla are in the D-sensilla field distal to the valve/flap on the epipharyngeal side; again, they comprise two, bilaterally symmetrical organs of five sensilla each. The two H-sensilla are each a separate gustatory organ because their neuron cell bodies and axons are in a separate plate in the head, the hypopharynx^11,18^.

Because of the incomplete list of precise descriptive terms for areas of the functional foregut on a fine resolution scale, researchers investigating *X. fastidiosa* biofilm spatial patterns are often left with ambiguous terms to describe biofilm locations, such as “proximal region of precibarium canal,” “medial region of the proximal precibarium on the epipharynx,” “area on the epipharynx with sutures,” and “opening into the cibarium”^11,22^. In particular, the epipharyngeal basin lacks precise descriptive terms. Its portions have been described as the “groove of the basin,” “top of the precibarial valve,” “top of the valve near the precibarial pit,” and “distal extremity of the precibarial valve”^11,22^. Furthermore, anatomical segments are often not clearly delimited in sharpshooter literature. Therefore, we developed new terms that consider biologically relevant delineations of the precibarial and cibarial structures to describe our 3D model of the functional foregut in *G. atropunctata*, particularly in the context of *X. fastidiosa* colonization.

Accordingly, the objectives of this paper are two-fold. First, we introduce and describe a new, 3D interactive anatomical model correlated with microscopy images. Second, the model is used to synthesize anatomical terminology for the complex parts of the functional foregut, with several proposed anatomical terms. Our larger goal is to better serve future multidisciplinary studies of *X. fastidiosa* acquisition, retention, and inoculation by sharpshooters.

## Results

### Illustration of the 3D model

**Figure 1:**
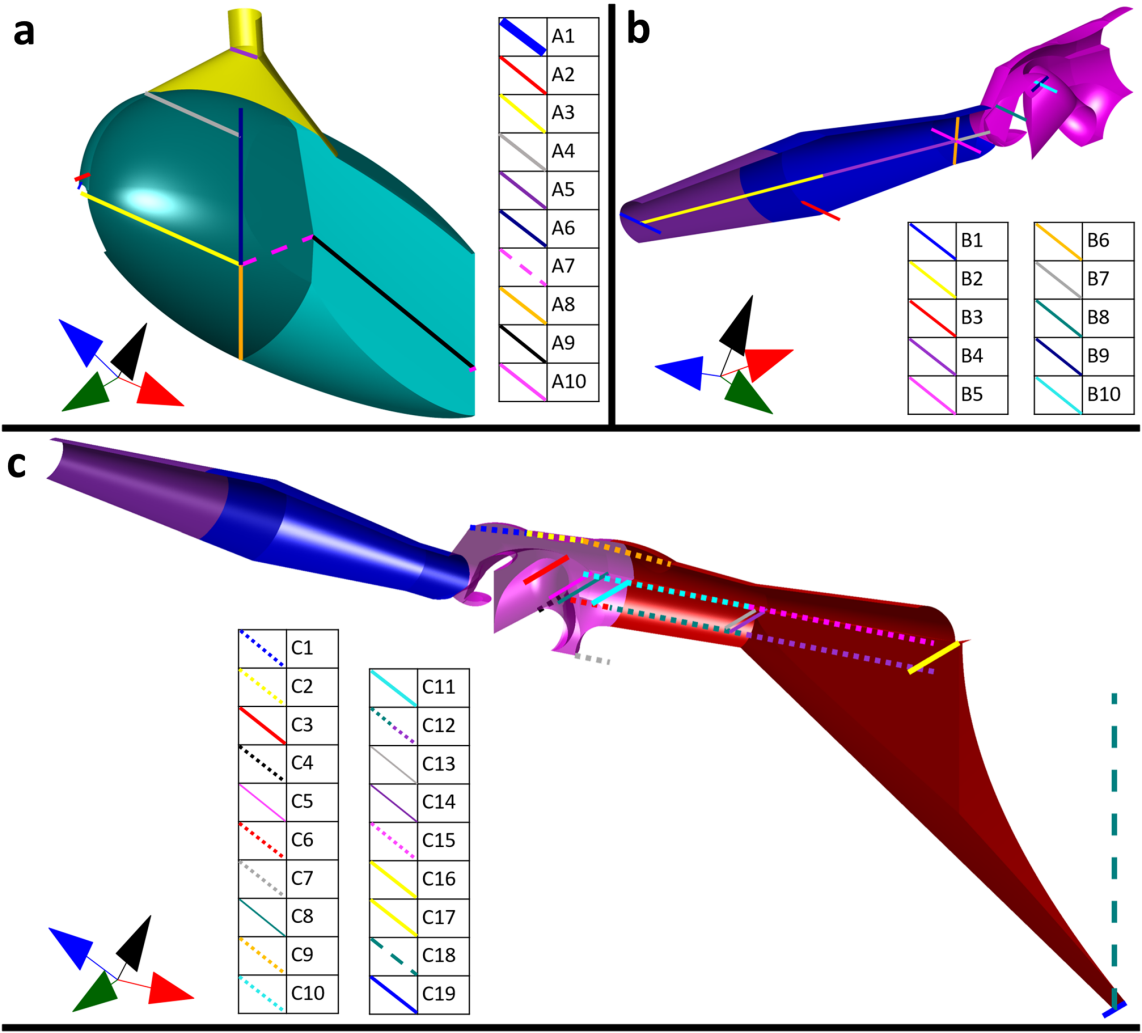
Dimensions of the 3D model for the functional foregut of the blue–green sharpshooter, as detailed in the Supplementary Information (Table S1). All views shown are internal (concave). (**a**) Left half of the true mouth (yellow) and cibarium (teal). (**b**) Left half of the HEF (purple), distal enclosure (blue), and basin (magenta). (**C**) Left half of the precibarium. The basin is partially translucent to make dimension C4 visible. The coordinate axes indicate anterior → posterior, distal → proximal (for the trough), ventral → dorsal, and left → right. The dimensions are indexed based on the subfigure in which they appear. For example, A7 is the seventh dimensional measurement that is illustrated in figure 1a. The 3D model segment color coding is explained in Figs. 2 and 3. HEF: hypopharyngeal extension that inserts into the stylet food canal.

**Figure 2:**
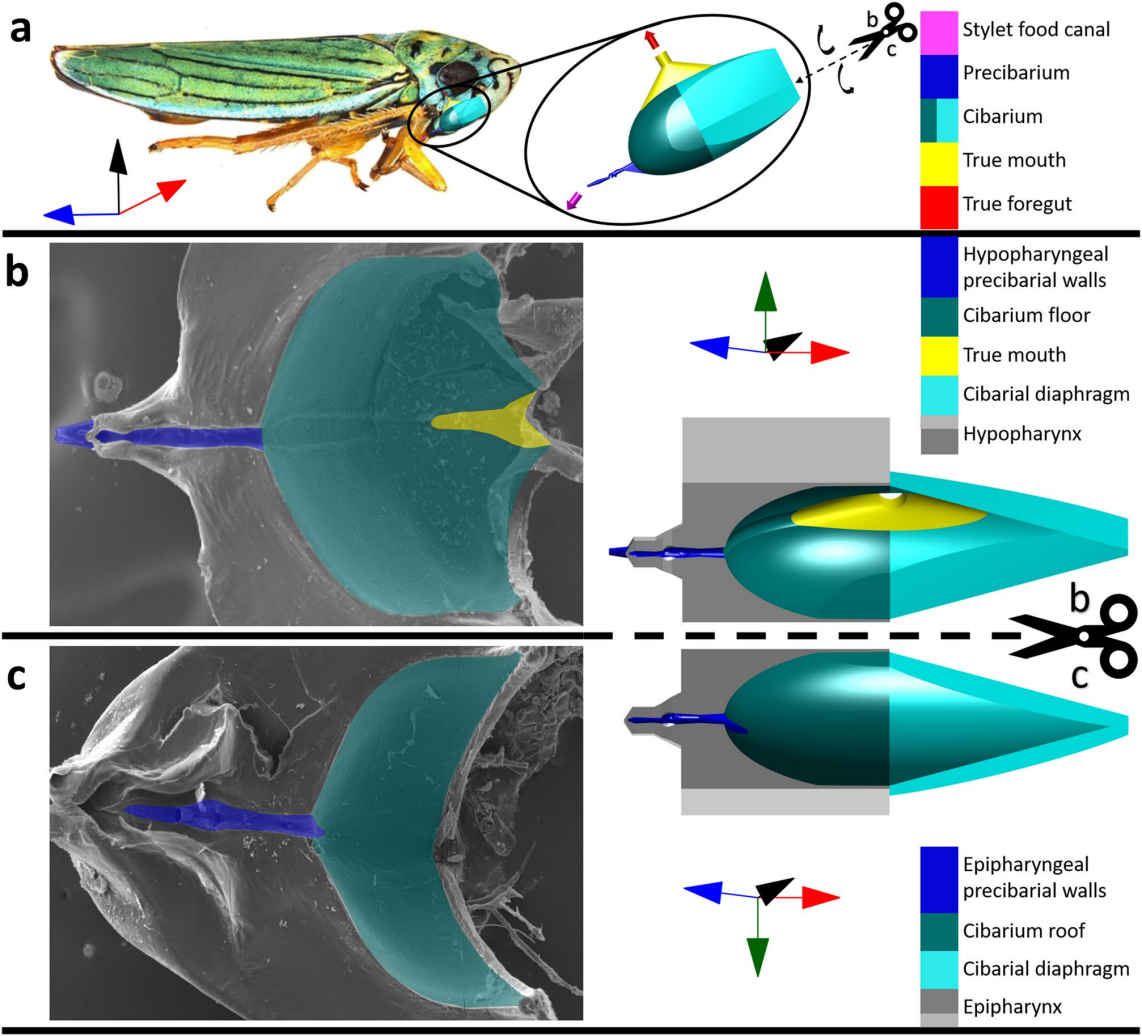
Functional foregut (precibarium and cibarium) of the blue–green sharpshooter, with segments of the 3D model labeled with broad terminology. (**a**) Approximate location/orientation of the functional foregut inside the insect. The 3D model shows an external view of the walls of the precibarium, cibarium, and true mouth, as if the rest of the epipharynx, hypopharynx, and cibarial diaphragm did not exist (convex). The image of the insect is used with the permission of the photographer Emilie Bess (EC Bess, USDA APHIS PPQ). (**b**) Hypopharynx electron microscope image (left) and 3D model segment (right, concave, internal) for comparison. The electron microscope image was adapted from a previous publication (Reprinted with permission; copyright © American Society for Microbiology, [Appl. Environ. Microbiol. 81, no. 23 (2015): 8145–8154 10.1128/AEM.02383–15]). (**c**) Epipharynx electron microscope image (left) and 3D model segment (right, concave, internal) for comparison. The coordinate axes indicate anterior → posterior, distal → proximal (for the trough), ventral → dorsal, and left → right. The relationship between the functional foregut and coordinate axes is different for different sharpshooter species. The scissors and dotted lines indicate where the 3D model in subfigure a would be split to obtain the 3D model segments in subfigures b and c. The associated black curved arrows in subfigure a indicate that the two halves of the split 3D model segment would need to be hinged outward to obtain the views in subfigures b and c. The gray 3D parts of subfigures b and c are semi-abstract representations of the epipharynx and hypopharynx that serve for comparison with their corresponding electron microscope images.

**Figure 3:**
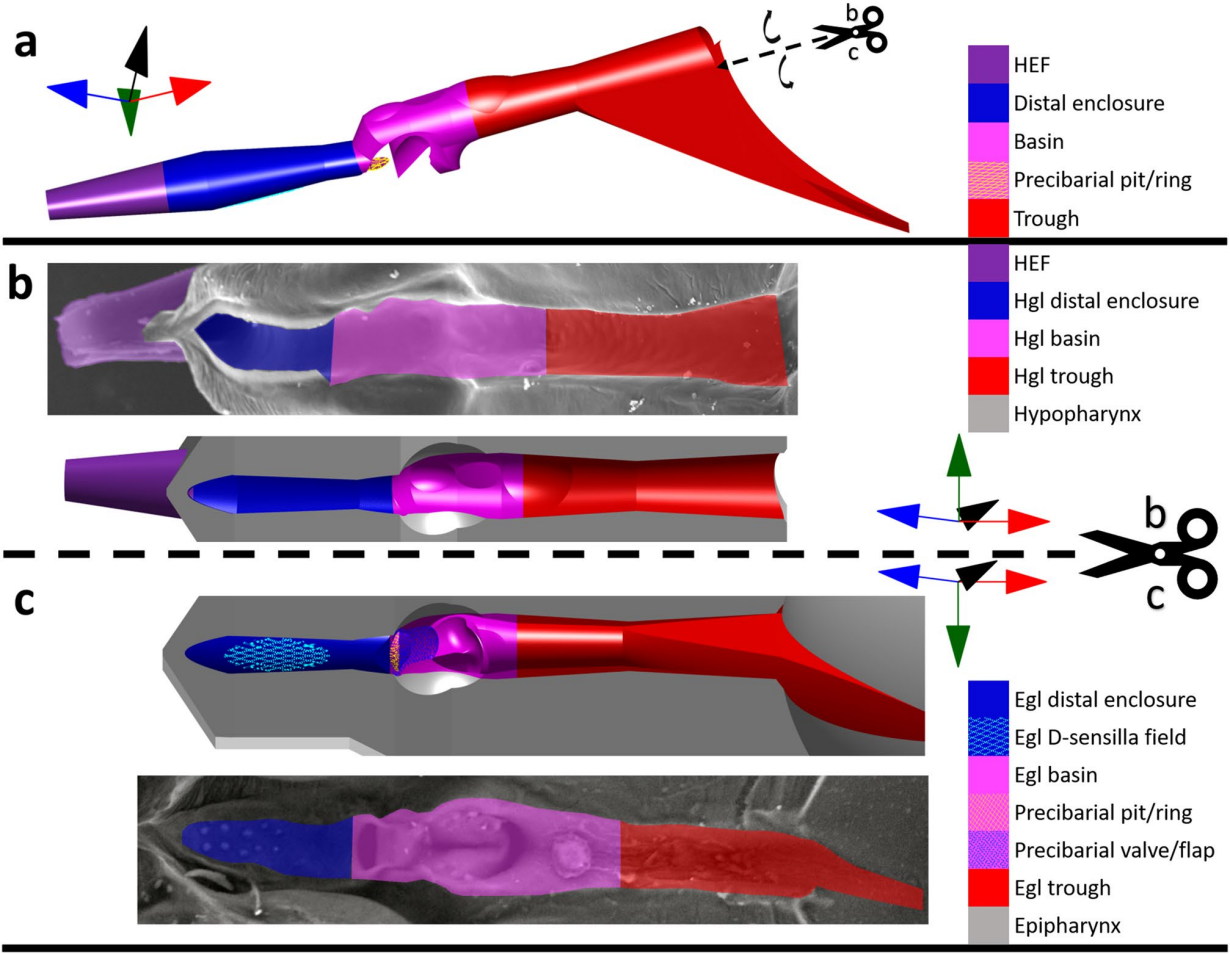
Close-up segments of the precibarium of the blue–green sharpshooter, labeled with broad terminology. (**a**) Precibarium 3D model segment. It shows an external view of the walls of the precibarium, as if the rest of the epipharynx and hypopharynx didn’t exist (convex). (**b**) Hypopharyngeal precibarial walls (colored, concave) in electron microscope image of a segment of the hypopharynx (top, internal) and equivalent view of the 3D model segment (bottom, concave) for comparison. (**c**) Epipharyngeal precibarial walls 3D model segment (top, mostly concave) and an equivalent electron microscope image (bottom, mostly concave) for comparison. The coordinate axes indicate anterior → posterior, distal → proximal (for the trough), ventral → dorsal, and left → right. The scissors and dotted lines indicate where the 3D model segment in subfigure a would be split to obtain the 3D model segments in subfigures b and c. The associated black curved arrows indicate that the two halves of the split 3D model segment would need to be hinged outward to obtain the views in subfigures b and c. The gray 3D parts of subfigures b and c are semi-abstract representations of the epipharynx and hypopharynx, which serve for comparison with their corresponding electron microscope images. Hgl: hypopharyngeal; egl: epipharyngeal; HEF: hypopharyngeal extension that inserts into the stylet food canal.

The 3D model mimics the curvature of the blue–green sharpshooter functional foregut with a precision absent in previous models. It incorporates complex morphologies and over 30 dimensions measured from published microscope images. The published microscopy images are of blue–green sharpshooters from different geographical regions and likely different sexes. Segments of the 3D model are illustrated in Fig. 1, together with the dimensions that are described in Supplementary Table S1. The dimensions are indexed based on the subfigure in which they appear. For example, A7 is the seventh dimensional measurement that is illustrated in Fig. 1a. The 3D model segment color coding is explained in Figs. 2 and 3.

The entire 3D model is illustrated in Fig. 2a. This view of the model has a different viewpoint than the concave microscope images from which it was derived (Supplementary Information, Table S1). The walls of the model represent walls of the precibarium, cibarium, and true mouth as they would be in undissected sharpshooters, where the hypopharynx and epipharynx are apposed and the cibarial diaphragm is attached to the cibarium bowl. Also, the 3D model is viewed from an imagined exterior of the functional foregut (convex), rather than the interior (concave). The approximate location of the 3D modeled anatomy in a blue–green sharpshooter is shown in Fig. 2a as if the viewer could see through the insect head. The cibarial diaphragm of the 3D model is fully extended as if the cibarial dilator muscles were fully contracted, pulling the cibarial diaphragm into the head to allow for complete filling of the cibarium. Figure 2a is used to define directional coordinate axes that are used in the rest of the 3D model figures. These coordinate axes are species-dependent, due to different orientations of the functional foregut in different sharpshooter species. For example, the 3D model of the functional foregut in Fig. 2a would need to be rotated around 30 degrees clockwise relative to the coordinate axes for these terms to be appropriately applied to the cibarium of the glassy-winged sharpshooter, *Homalodisca vitripennis* (Germar, 1821).

If the 3D model were split open and its two halves hinged outward, its relation to microscopy images would be more intuitive, as shown in Fig. 2b, c. In these subfigures, semi-abstract 3D representations of the epipharynx and hypopharynx (gray in the figures) are illustrated together with the 3D model halves on the right side for comparison with their corresponding SEM images on the left side. Segments of the model halves are shown as concave depressions in the epipharynx and hypopharynx. The adjacent SEMs show the natural, concave view of the functional foregut structure. They are color coded to correspond with color coding in the 3D model halves.

Figure 2 is useful for understanding how the epipharynx, hypopharynx, and cibarial diaphragm form the walls of the 3D model. Figure 3 is similar to Fig. 2, except it focuses on the precibarium. In Fig. 3a, the precibarial segment of the 3D model in Fig. 2a is shown up close from an external view as a convex tube-like structure. In the following subfigures, the 3D precibarium exterior is split into its two halves and hinged outward. The concave halves of the precibarium are illustrated as depressions in the semi-abstract representations of the epipharynx and hypopharynx. The four main segments of the precibarium are color coded in both the 3D model and the corresponding SEMS.

The terminology illustrated in Figs. 2 and 3 explains our 3D model and is also helpful for gaining a broad sense of sharpshooter functional foregut anatomy. Figure 2 illustrates how the cibarium roof in blue–green sharpshooters is more ventral than the cibarium floor, which is counter-intuitive for non-entomologists because of the opisthognathous (posteriorly directed) orientation of mouthparts in leafhoppers. Figure 3 illustrates how the precibarium is broadly made of four segments, one of which is previously un-named. We propose the name “distal enclosure” for the previously un-named region of the precibarial walls that includes the D-sensilla field (Fig. 3a–c). This is because the term “D-sensilla field” is an abbreviation for “distal precibarial chemosensilla field” and having a shorter name for this segment will make it easier to describe.

### Morphological accuracy of the 3D model

Figures 1, 2 and 3 are supported by our 3D interactive figures in the Supplementary Information. The interactive figures are helpful for gaining an intuitive understanding of the 3D model morphology, including the concavity of the model when viewed from different perspectives. The model is designed for a balance between precision of fluid dynamic modeling and time costs associated with creating and using a complex 3D model.

**Figure 4:**
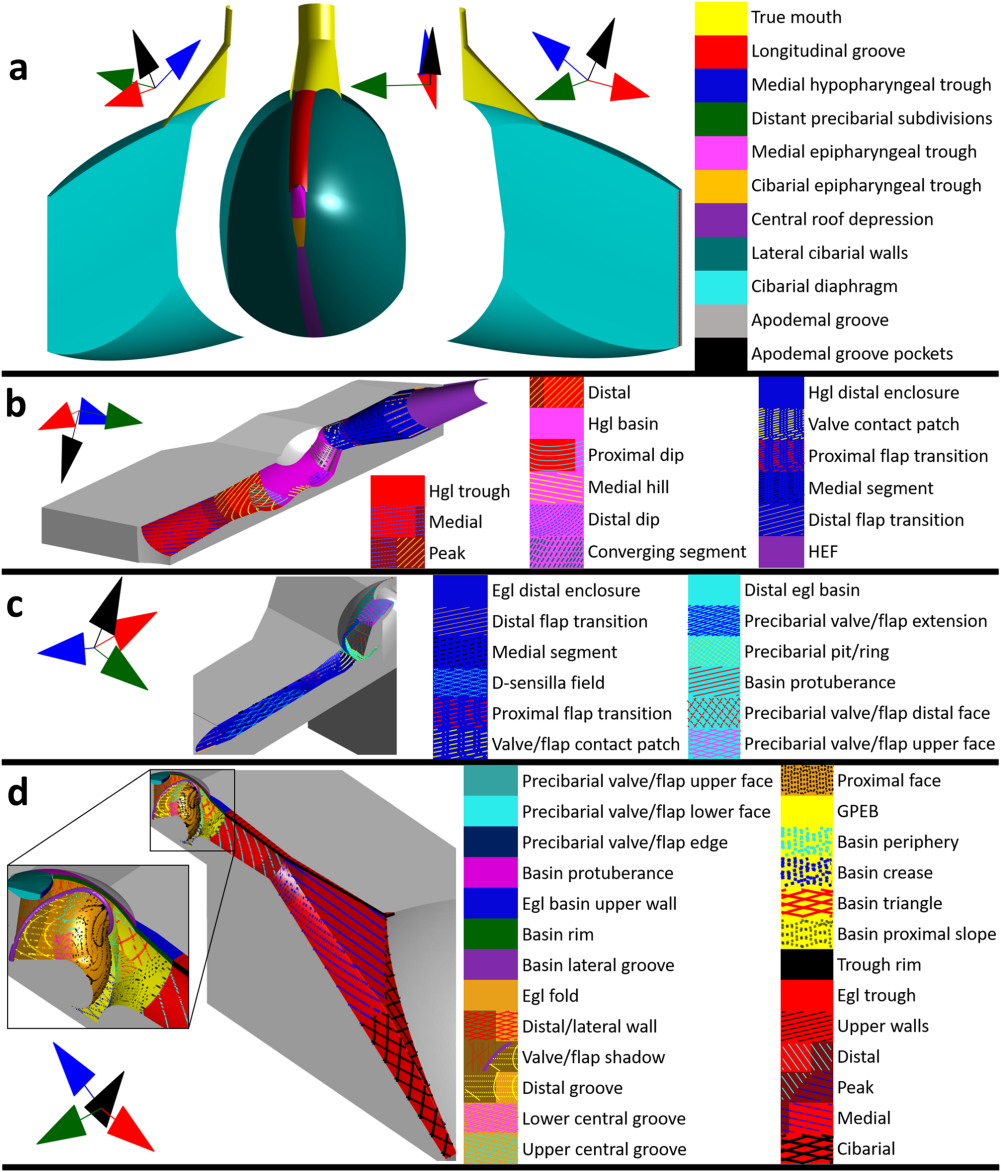
Fine resolution terminology for the cibarium and precibarium of the blue–green sharpshooter. In this figure, all model views are internal. (**a**) Cibarium bowl (concave, with the cibarial diaphragm and true mouth split outward, like classic plantation shutters (concave). (**b**) Left half of the hgl precibarium (concave). (**c**) Left half of the egl distal enclosure (concave) and distal egl basin (partially concave and partially convex). (**d**) Left half of the egl trough and part of the egl basin (the egl fold and basin protuberance are convex, and the rest is concave). The egl fold lateral groove was modified for the sake of illustration. The coordinate axes indicate anterior → posterior, distal → proximal (for the trough), ventral → dorsal, and left → right. The gray 3D parts of subfigures b–d are abstract representations of the epipharynx and hypopharynx. Hgl: hypopharyngeal; egl: epipharyngeal; GPEB: groove at the proximal end of the basin; HEF: hypopharyngeal extension that inserts into the stylet food canal.

The model incorporates morphological features that are important for fluid dynamics studies, while ignoring morphologies that are less important for such studies. The primary example of this is the shape of the cibarium model, particularly the ratio of the cibarium floor radius to the cibarium roof radius (Fig. 1, Dimensions A6 and A8). This ratio is 1.7 in our model, based on a previously published microscope image that shows a blue–green sharpshooter cibarium bowl from a viewpoint similar to that in Fig. 4a ^6^ However, micro-CT measurements in a recently published study show a ratio that is closer to 2.2^12^. See Supplementary Fig. S1. The discrepancy between these ratios appears to be due to biological variability, possibly based on the geographically different populations from which blue–green sharpshooters were obtained. Thus, future studies with larger insect sample sizes than those available for the meta-analysis would be helpful in determining the extent to which cibarium fluid dynamics are affected by cibarium shape variability. In contrast, blue–green sharpshooter precibarium length appears to have smaller variability, with a reported standard deviation of only 6.9%^12^.

Our model of the precibarium is more precise than that of the cibarium. For example, the view of the egl basin in Fig. 1c reveals a segment under the precibarial valve/flap that is shaped like a triangle pointing downward. This triangular segment represents the inward extension of the basin central groove, which is inferred to be deeper toward the precibarial valve/flap. The segment of the model to the right of the downward pointing triangle, which also protrudes below the rest of the basin, is part of the groove at the proximal end of the basin. While the shapes of this groove and the basin central groove are not perfectly accurate, they serve to account for stagnation zones when fluid flows in and out of the precibarium. Similarly, the precibarial pit/ring (Fig. 3a, c) serves as a pocket with an imprecise shape that accounts for a predicted stagnation zone.

The precibarial trough segment (Fig. 3a) exhibits the essential widening toward the cibarium, while ignoring the flared shape that is present in SEMs (Fig. 3b, c). This will allow for order of magnitude predictions of fluid drag forces, while avoiding the time and computational costs of designing a 3D model that flares toward the cibarium. In these ways, the 3D model balances complexity with cost.

The precibarium is speculated to be the portion of the foregut where most bacterial detachment occurs during insect feeding. Thus, it is appropriate that the precibarial segment of our 3D model was constructed with more attention to fine-resolution details, allowing for more precise estimation of fluid dynamics in this segment in future studies. In the following section, the morphology of the 3D model, particularly in the precibarium, is cross-referenced with the corresponding anatomical segments observed in the SEMs.

### Fine-scale spatial-resolution terms for the precibarium and cibarium

**Figure 5:**
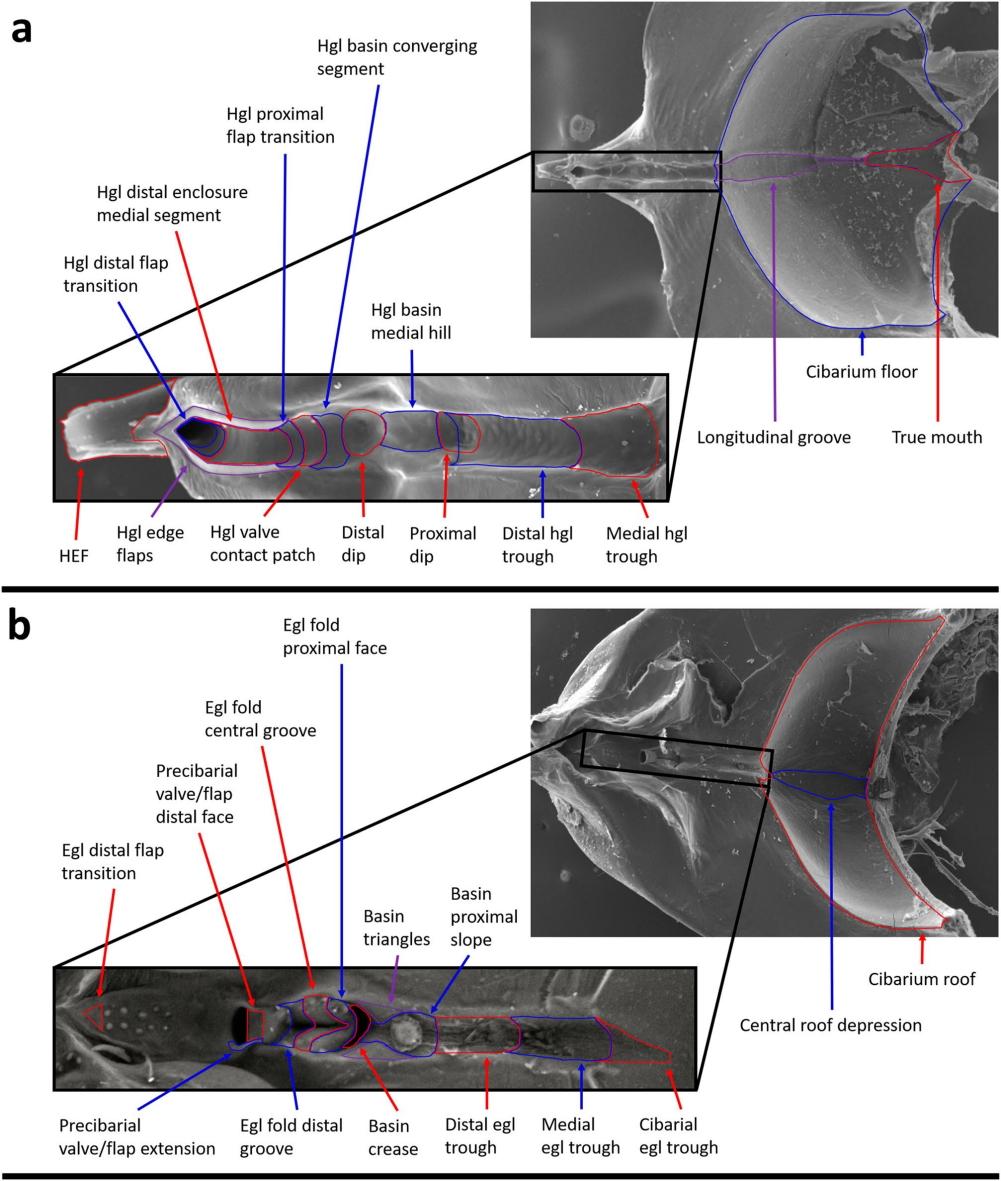
Fine resolution terminology for the precibarium and cibarium of the blue–green sharpshooter, labeled using scanning electron micrographs. (**a**) Hypopharynx. The electron microscope image on the top right was adapted from a previous publication (Reprinted with permission from Rapicavoli et al. 2015; copyright © American Society for Microbiology, [Appl. Environ. Microbiol. 81, no. 23 (2015): 8145–8154 10.1128/AEM.02383–15]). (**b**) Epipharynx. hgl: hypopharyngeal; egl: epipharyngeal; HEF: hypopharyngeal extension that inserts into the stylet food canal.

We present in Figs. 4 and 5 finer resolution terminology than described in the Introduction and new terms intended to aid in describing biofilm spatial patterns and fluid dynamics. When new terminology is introduced, it is denoted as ‘proposed herein’ (PH).

In Fig. 4a, the anterior/dorsal view of the cibarium bowl reveals an alternative way of describing cibarium segments that are illustrated in Fig. 2. The cibarium bowl can be divided into the longitudinal groove, central roof depression (PH), and lateral cibarial walls (PH) instead of the cibarium roof and cibarium floor. Figure 4a also reveals a portion of the precibarium: the cibarial epipharyngeal trough (PH), medial epipharyngeal trough (PH), and medial hypopharyngeal trough (PH) are visible (see also Fig. 5).

The hypopharyngeal (hgl) precibarial wall segments presented in Fig. 3b can be subdivided (Figs. 4b, 5a). The hgl trough is composed of the medial and distal subdivisions (PH), which meet at the hgl trough peak (PH). The proximal dip (PH) straddles the hgl trough and hgl basin, which have boundaries that are based on the boundaries of the epipharyngeal trough and epipharyngeal basin. The hgl basin contains the medial hill, distal dip, and converging segments (PH). The hgl basin meets the hgl distal enclosure at the distal edge of the converging segment.

The hgl distal enclosure is composed of the hgl valve/flap contact patch (PH), hgl proximal flap transition (PH), hgl medial segment of the distal enclosure (PH), and hgl distal flap transition (PH). The hgl valve/flap contact patch is the portion of the wall that remains bare of *X. fastidiosa* after a shag carpet-like biofilm develops. The hgl proximal flap transition is demarcated by the region where the hgl edge flaps (PH) transition to their full width. The hgl medial segment of the distal enclosure is demarcated by the region where the hgl edge flap widths and the distance between the left and right edge flaps remain relatively constant. The hgl distal flap transition is demarcated by the region where the distance between the left and right hypopharyngeal edge flaps decreases, making the shape of a triangle.

The epipharyngeal (egl) precibarial wall segments presented in Fig. 3c can also be subdivided (Figs. 4c, d, 5b). The egl trough is composed of the cibarial, medial, distal, upper (dorsal/posterior) walls, and rim subdivisions (PH). The cibarial and medial subdivisions are separated by an imaginary plane where the rim of the trough meets the cibarium that is perpendicular to the precibarial trough centerline. The medial and distal subdivisions meet at the egl trough peak (PH).

The egl basin is divided into five segments (Figs. 4c, d, 5b): (1) the groove at the proximal end of the basin, (2) the egl fold (PH), (3) the rim of the basin, (4) the egl basin upper (dorsal/posterior) walls (PH), and (5) the distal egl basin (PH). The groove at the proximal end of the basin (GPEB) contains the triangles, proximal slope, crease, and periphery segments (PH). The basin proximal slope is the segment of the GPEB proximal to the basin triangles, which is shaped like a rounded groove of a pulley. The proximal end of the basin proximal slope is demarcated by a transition from the tubular shape of the distal egl trough to the shape of a pulley groove. The distal end of the basin proximal slope is demarcated by a reversal in the concavity of the GPEB wall. The remaining portions of the GPEB walls form the crease and periphery segments. In our 3D model, the basin periphery is mostly in the space between the basin triangles and egl fold (PH). The boundaries of the GPEB were altered from their original limits herein to allow for more precise and simplified naming conventions.

The egl fold (Figs. 4d, 5b) contains the proximal face (PH), central groove, distal groove (PH), distal/lateral wall (PH), and lateral groove (PH). The central groove is demarcated proximally by the edge of the rounded proximal face. It is demarcated distally by the widening of the distal groove. The central groove contains lower (ventral) and upper (dorsal) segments (PH), demarcated by the opening of the central groove. The distal groove contains a region that remains hidden behind the precibarial valve/flap in dorsal/distal views of the egl basin. This region is the valve/flap shadow (PH). The distal/lateral wall of the egl fold is composed of wall segments that may form a concave shape at the distal and lateral ends of the epipharyngeal fold. The lateral groove separates the distal/lateral wall from the remainder of the egl fold.

The distal egl basin contains the basin protuberances (PH), the precibarial valve/flap, and the precibarial pit/ring. The precibarial valve/flap contains the lower (ventral) face, edge, upper (dorsal) face, distal face, and extension segments (PH). The precibarial valve/flap extension begins at the distal edge of the upper face and wraps around the sides of the precibarial pit/ring.

At the distal edge of the precibarial pit/ring, the distal egl basin meets the egl distal enclosure. The egl distal enclosure is composed of the egl valve/flap contact patch (PH), egl proximal flap transition (PH), egl medial segment of the distal enclosure (PH), and egl distal flap transition (PH). The boundaries of these segments are delimited by their apposed segments on the hypopharynx. The egl medial segment of the distal enclosure includes the D-sensilla field.

## Discussion

The understanding of hemipteran feeding anatomy and physiology has advanced greatly since the time of Snodgrass (1935), an original authority for insect anatomy, principally through development of new technologies like electron and confocal microscopy and EPG. In-depth anatomical descriptions and terminology has often not kept pace. Herein, our goal was to remedy this problem and provide a foundation for future fluid dynamics studies. For example, the term ‘functional foregut’ was recently enlarged to include the food canal, because fluid flow is continuous through the stylet food canal, precibarium, and cibarium^10,23^. However, in this paper and future ones, we recommend reverting back to the earlier definition because the foregut is internal to the head of an insect^19^, while the mouth parts are not. Also, although there are anatomical synonyms for the functional foregut (e.g., the buccal cavity and preoral cavity), here we exclusively used functional foregut because it emphasizes functionality and is commonly referenced in the *X. fastidiosa*-sharpshooter literature^12,20,23–25^.

The foregut functionality we highlight is that of fluid movements, which are highly dynamic in this area of the complete foregut. Fluids can be taken up into the functional foregut, moved around, then either ejected (termed egestion) or separate boluses of fluid can be swallowed past the true mouth. Egestion and ingestion are the fluid movements that are most important for *X. fastidiosa* inoculation and acquisition, respectively. While *X. fastidiosa*-like bacteria have been observed in both the stylet food canal and true foregut, colonization patterns have been described mainly in the precibarium and cibarium of sharpshooters^6,7,11,15,16^. Studies and hypotheses to date support that the cibarial dilator and precibarial valve muscles provide the sole propulsive power to cause egestion and concomitant inoculation of bacteria that have colonized the precibarium and cibarium^19,20^. There is no evidence that bacteria can be inoculated from the true foregut, which is not included in our 3D model.

Recent SEM observations of the spittlebug, *Philaenus spumarius*^26^, revealed precibarial anatomy and lumenal structures nearly identical to those of sharpshooters. There are two sets of gustatory organs separated by the precibarial valve, which can open via action of a tiny precibarial valve muscle. The arrangement facilitates two-stage tasting of fluids brought into the precibarium. This similarity in separation of gustatory organs is not surprising, because such an orientation has been found in all hemipterans examined[^27^, E.A.B. unpub. data,^28^^,^^29^]. Extensive light microscopy and transmission electron microscopy of the precibarial valve interior of spittlebugs by Ruschioni et al. (2019) revealed a previously undescribed bell-like invagination whose opening is the precibarial pit (termed ring in that paper). The precibarial valve muscle is attached to the bell-shaped invagination in spittlebugs, unlike the proposed attachment directly to the pit/ring and flap in sharpshooters, as hypothesized by Backus and McLean (1982). Thus, while the flap was considered the actual valve by Backus and McLean (1982), Ruschioni et al. (2019) call the entire assembly the valve, with the flap only a part thereof. It is not yet known whether the lumenal structures of sharpshooters have similar internal anatomy to those of spittlebugs, despite their similar external, lumenal appearances. Because of this terminological uncertainty, herein we referred to the precibarial valve/flap until the interiors of sharpshooter structures are studied using transmission electron microscopy.

In the past, broad understanding of sharpshooter functional foregut anatomy was not always sufficiently detailed for studies of the involvement of precibarial structures in *X. fastidiosa* acquisition and inoculation. Biofilm spatial patterns in the precibarium and cibarium are nuanced and have a finer resolution than the originally applied anatomical understanding^7,11,15^. Our study will allow for higher resolution descriptions of biofilm spatial patterns and fluid dynamics.

In conclusion, we had two objectives for this work. First, we introduced the 3D model, which mimics geometric complexity of the auchenorrhynchan/sharpshooter hemipteran functional foregut with finer spatial resolution than any previous model. The model is based on published microscopy images of blue–green sharpshooters from different geographical regions and likely different sexes. Second, we used the 3D model to synthesize the existing anatomical understanding of the sharpshooter foregut and propose new terminology, equipping multidisciplinary researchers to become proficient in anatomical details. These details will provide finer resolution and delimited boundaries to aid in describing the precise locations of *X. fastidiosa* biofilm formation sites. Both our new 3D model and the enhanced anatomical understanding will be helpful for future fluid dynamic studies of the functional foregut, which have essentially trended in the direction of determining flow characteristics with increasing spatial resolution^6,12,14,26^. An enhanced ability to model fluid dynamics and describe biofilm spatial patterns in blue green sharpshooters will allow us to evaluate how physical forces affect *X. fastidiosa* acquisition, retention, and inoculation. Increased understanding in these areas could lead to new targets for preventing diseases caused by *X. fastidiosa*.

## Methods

### 3D model construction

A 3D model of the blue–green sharpshooter functional foregut (and true mouth) was created using COMSOL Multiphysics (COMSOL, Inc., Stockholm, Sweden) based on a meta-analysis of previously published microscopy images of sharpshooters. The analyzed microscopy images were of insects from different geographical regions and likely different sexes. The model incorporates over 30 dimensions collected from the literature (Supplementary Information, Table S1). The 3D model dimensions are consistent with those of Ranieri et al., as described in the Supplementary Information^12^.

Segments of the model were isolated and meshed in COMSOL Multiphysics. Meshes were exported as STL files that were interpreted in MATLAB (MathWorks, Inc., Natick, MA) as triangulations. Triangulations were displayed in Figs. 1, 2, 3, 4 and 5 using content from MATLAB Central’s File Exchange^30–32^.

Interactive versions of the 3D models in Figs. 1, 2 and 3 were created as supplementary figures in a PDF file. Interactive figures were created using MATLAB, Adobe Acrobat Pro DC (San Jose, CA), content from GitHub, and content from MATLAB Central’s File Exchange^30,31,33–37^. The interactive figures do not contain measuring lines (Fig. 1) or hatching (Fig. 3). This is because of challenges involved with converting these types of content into a U3D file format, which was used for creating the interactive figures. The interactive figures are accessible on computers using Adobe Acrobat Reader DC.

### Scanning electron microscopy

The morphology in this article is illustrated with a mix of previously published and unpublished scanning electron micrographs from the Rapicavoli et al. (2015) study. Detailed methods can be found therein. In brief, blue–green sharpshooter heads were dehydrated and critical point dried using a series of ethanol dilutions. Functional foreguts were dissected from insect heads, mounted, coated with a platinum-palladium mixture, and imaged using a Philips XL30 field emission gun (FEG) scanning electron microscope (FEI, Hillsboro, OR).

### Directional terms

Directional terms are illustrated using the coordinate axes in Fig. 2a. Anterior and posterior point toward the space in front of and behind the insect, respectively. Note that, unlike most insects, sharpshooters (like other leafhoppers) have opisthognathous heads; that is, they are affixed under the head and pointed backwards. Thus, the mouthparts are unintuitively oriented posterior-ward (Fig. 2a). Ventral and dorsal point toward the space below and above the insect in Fig. 2a, respectively. Proximal and distal indicate a position that is close to and distant from an orientation site, respectively, and are used to describe progression and regression (respectively) along the ingestion route through the stylet food canal, functional foregut, and true mouth^20^. Also, left and right are used to indicate direction from the insect’s perspective in later subfigures.

Another important aspect of the fine resolution terminology is its cross-applicability to sharpshooter species besides the blue–green sharpshooter. Despite defining directional terms above, anatomical names that include the words “posterior,” “anterior,” “dorsal,” and “ventral” are avoided herein because the relationship between the functional foregut orientation and these directional terms varies for different sharpshooter species. For example, the 3D model of the functional foregut in Fig. 2a would need to be rotated around 30 degrees clockwise relative to the coordinate axes for these terms to be appropriately applied to the cibarium of the glassy-winged sharpshooter, *Homalodisca vitripennis* (Germar, 1821). If blue–green sharpshooter anatomy were labeled using coordinate axes that vary between species, then studies of *X. fastidiosa* colonization patterns that compare multiple sharpshooter species could be unnecessarily complicated. Therefore, the terminology proposed herein uses the terms “upper” and “lower” where the terms “posterior,” “anterior,” “dorsal,” and “ventral” could not otherwise be avoided.

## Supplementary Information

Supplementary Information 1.

Supplementary Information 2.
