# Supplementary material for: Functional foregut anatomy of the blue–green sharpshooter illustrated using a 3D model
Source: Sci Rep. 2021 Mar 22;11:6536. doi: 10.1038/s41598-021-85954-4 (PMC7985137; doi:10.1038/s41598-021-85954-4)

Interactive 3D Figures

Functional Foregut Anatomy of the Blue-green Sharpshooter Illustrated Using a 3D Model

This supporting material contains interactive 3D versions of Figs. 1, 2, and 3. It may take around a minute for each subfigure to activate after clicking on it and giving permission to view content. If there is trouble viewing the details of the model, try changing the lighting.

Fig. 1a

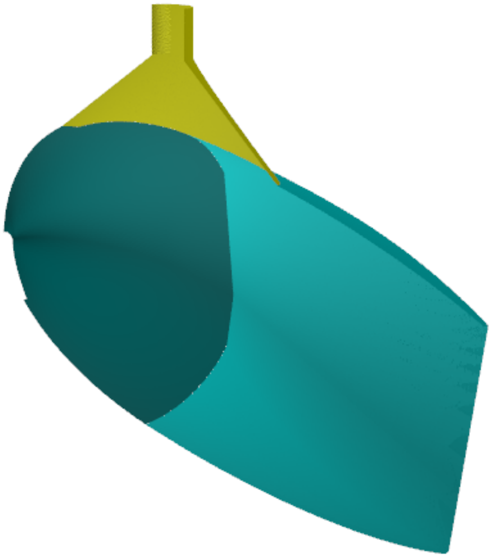

Fig. 1b

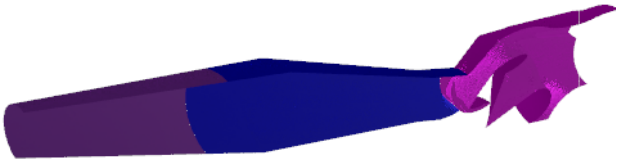

Fig. 1c

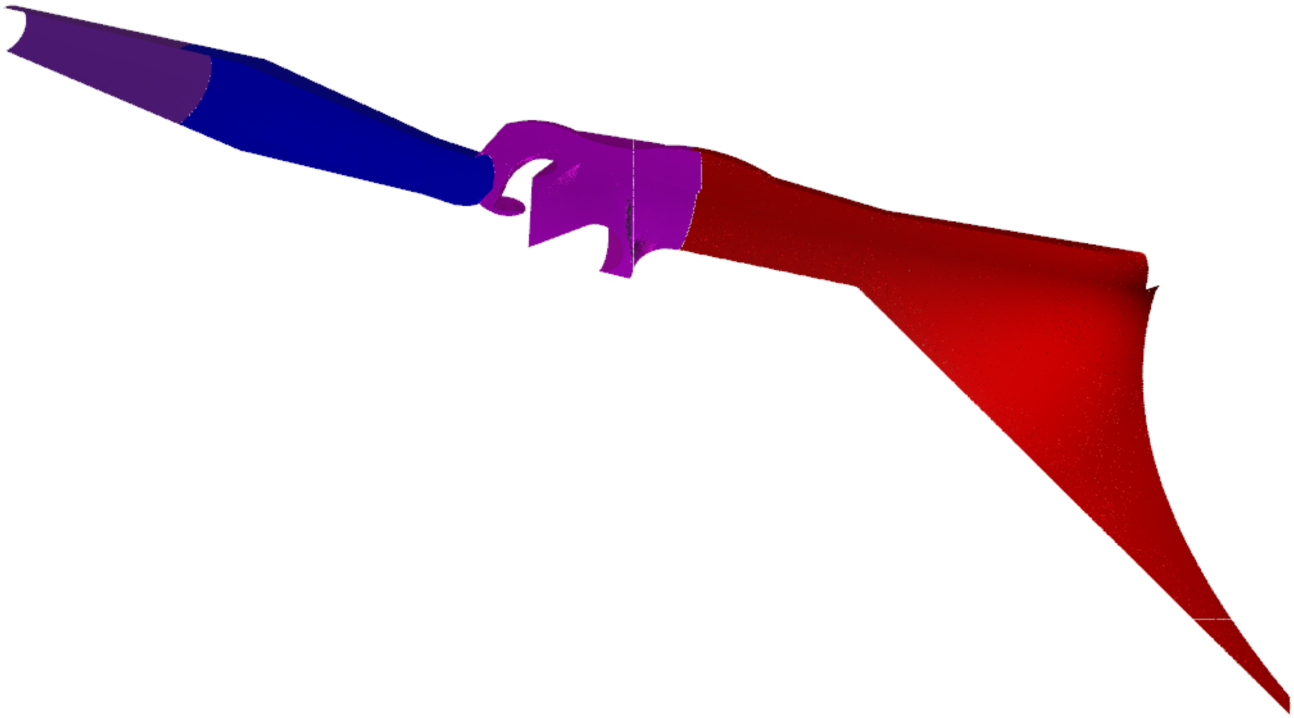

Fig. 2a

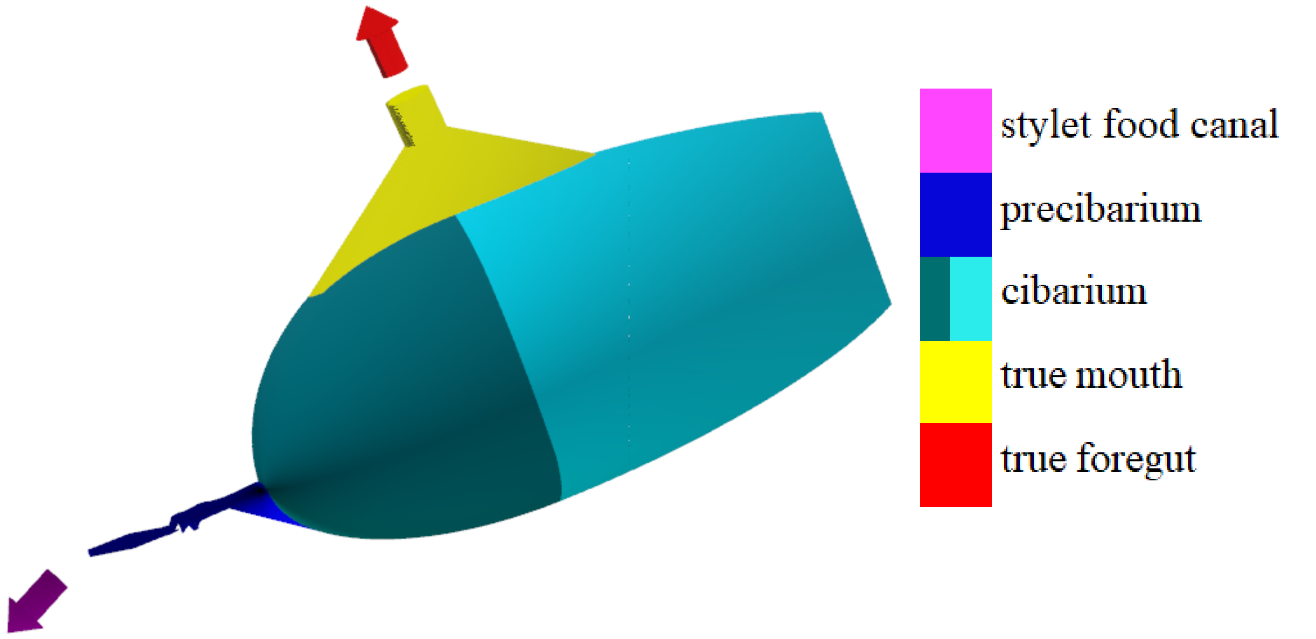

Fig. 2b

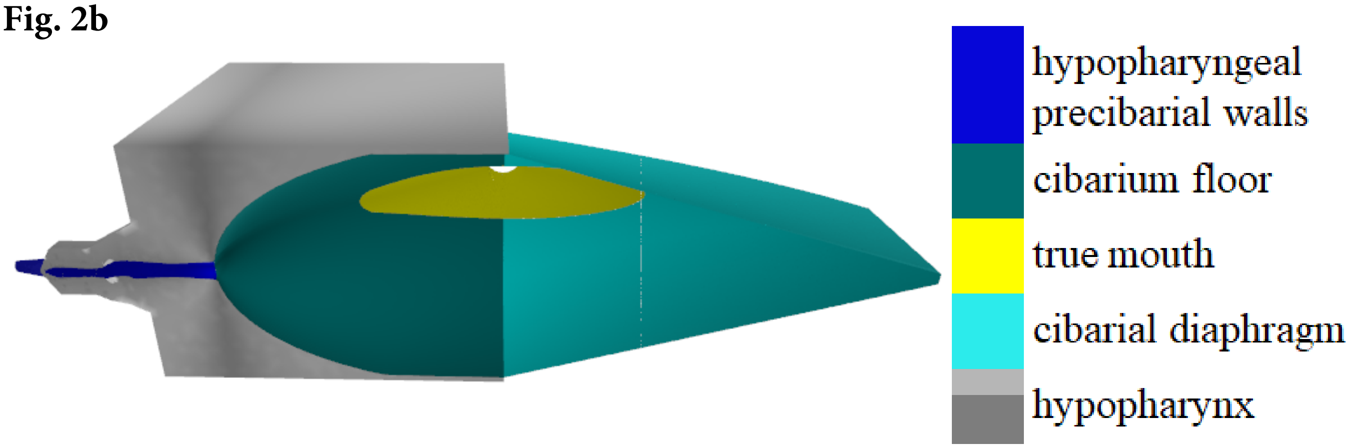

Fig. 2c

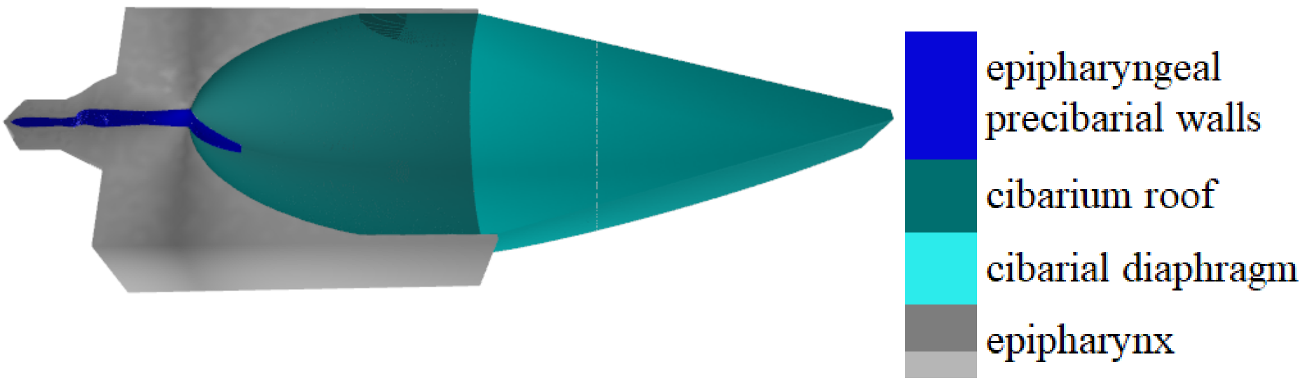

Fig. 3a

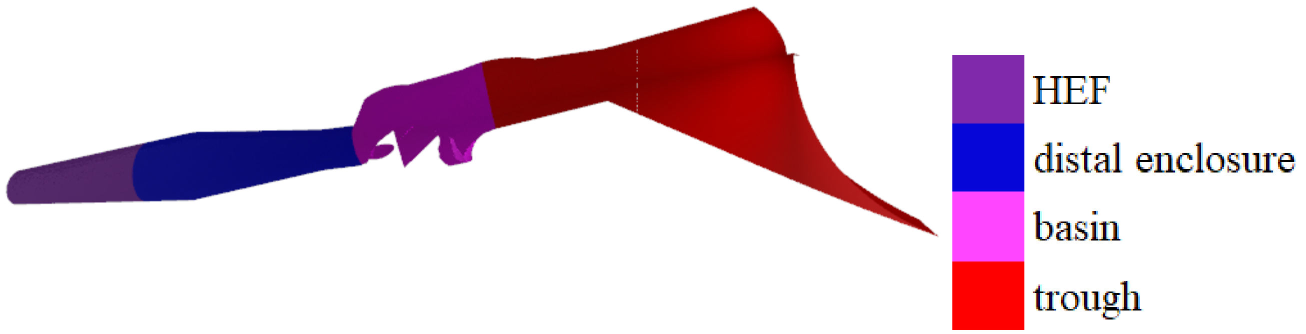

Fig. 3b

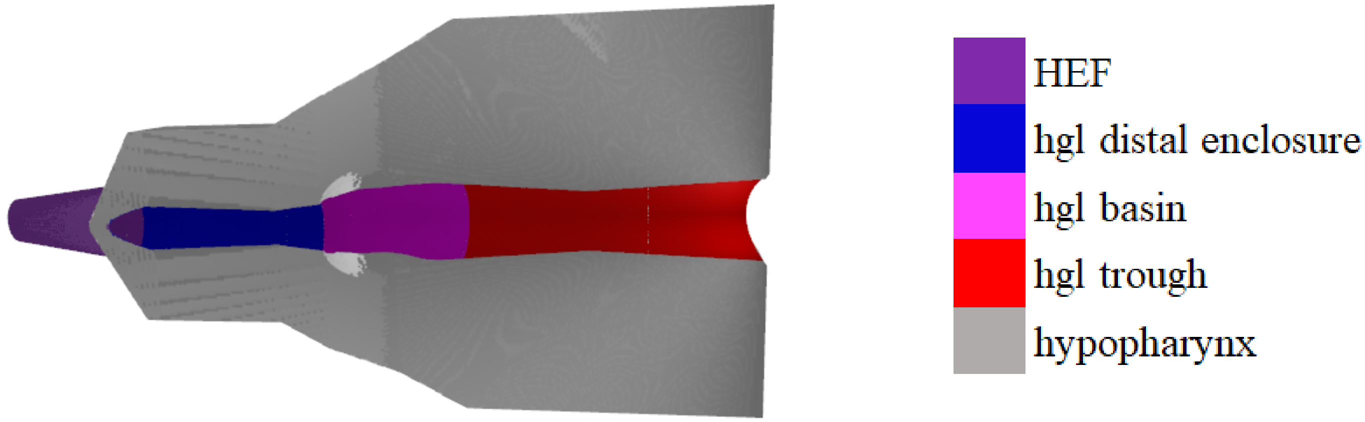

Fig. 3c

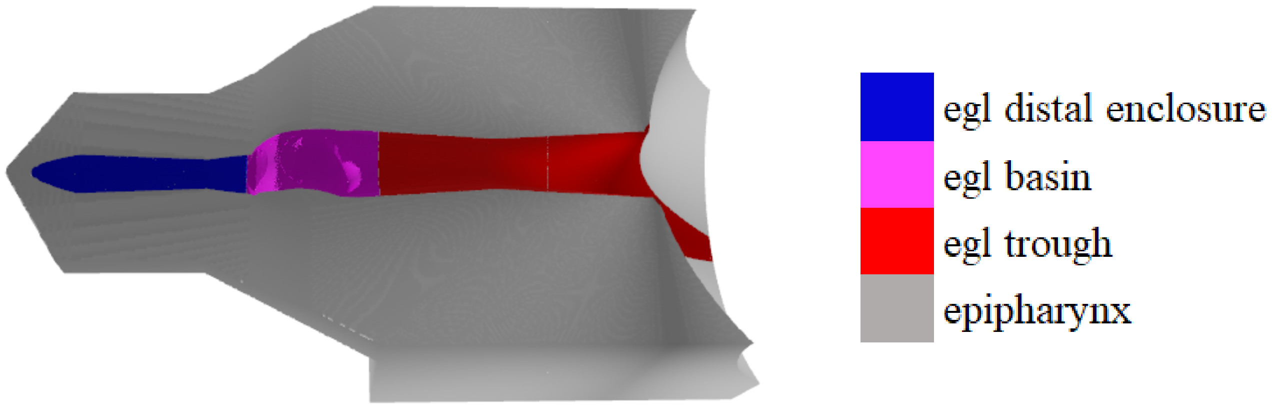

Supplement: Supplementary file 1 — Supplementary Information 1. [file 41598_2021_85954_MOESM1_ESM.pdf]
