# Supplementary material for: Functional foregut anatomy of the blue–green sharpshooter illustrated using a 3D model
Source: Sci Rep. 2021 Mar 22;11:6536. doi: 10.1038/s41598-021-85954-4 (PMC7985137; doi:10.1038/s41598-021-85954-4)
Supplement: Supplementary file 2 — Supplementary Information 2. [file 41598_2021_85954_MOESM2_ESM.docx]

Supplementary Information for

Functional Foregut Anatomy of the Blue-green Sharpshooter Illustrated Using a 3D Model

Daniel White^1^, Elaine A. Backus^2,5,6^, Ian M. Marcus^3^, Sharon L. Walker^1,3^ and M. Caroline Roper^4, *^

^1^ University of California, Riverside, Department of Chemical and Environmental Engineering, A220 Bourns Hall, 900 University Ave, Riverside, CA 92521

^2^ United States Department of Agriculture, Agricultural Research Service, San Joaquin Valley Agricultural Sciences Center, 9611 South Riverbend Avenue, Parlier, CA 93648-9757, U.S.A.

^3^ Drexel University, Department of Civil, Architectural, and Environmental Engineering, 3141 Chestnut Street, Philadelphia, PA 19104

^4^ University of California, Riverside, Department of Microbiology and Plant Pathology, 900 University Ave, Riverside, CA 92521

^5^ Mention of trade names or commercial products in this publication is solely for the purpose of providing specific information and does not imply recommendation or endorsement by the U.S. Department of Agriculture. USDA is an equal opportunity provider and employer.

^6^ This article was prepared by a U.S. Department of Agriculture employee as part of his/her official duties. Copyright protection under U.S. Copyright Law Title 17 U.S.C. § 105 is not available for such works. Accordingly, there is no copyright to transfer. The fact that the private publication in which the article appears is itself copyrighted does not affect the material of the U.S. Government, which can be freely reproduced by the public. Articles and other publications prepared as part of a Federal employee’s official duties are property of the U.S. Government.

**Table S1:** Dimensions of the 3D model of the blue-green sharpshooter functional foregut, as illustrated in Fig. 1. Dimensions were estimated based on published microscopy images of blue-green sharpshooters, except where Alves et al. (2008) was cited. hgl: hypopharyngeal; egl: epipharyngeal; HEF: hypopharyngeal extension that inserts into the stylet food canal.

| **#** | **Anatomical Feature** | **Length (µm)** | **References used to estimate length** |
| --- | --- | --- | --- |
| A1 | Longitudinal groove depth | 4 | (Purcell et al. 1979) |
| A2 | Longitudinal groove width | 26 | Longitudinal grove width in the 3D model is around 26 µm (Rapicavoli et al. 2015). |
| A3 | Cibarium length radius | 290 | (Rapicavoli et al. 2015) |
| A4 | True mouth length radius | 0.6*cibarium length radius | (Alves et al. 2008) |
| A5 | True mouth cylinder diameter | 50 | (Alves et al. 2008) |
| A6 | Cibarium floor radius | 215 | (Purcell et al. 1979) |
| A7 | Cibarium lateral radius | 124 | Estimated as 137 µm, but the dimension is 124 µm in the 3D model (Purcell et al. 1979 , Rapicavoli et al. 2015) |
| A8 | Cibarium roof radius | 130 | (Purcell et al. 1979) |
| A9 | Cibarial diaphragm side wall length | 1.5*cibarium length radius | (Alves et al. 2008) |
| A10 | Apodemal groove width | 11 | (Purcell et al. 1979) |
| B1 | HEF distal lateral diameter | 12 | (Purcell and Almeida 2003 , Almeida and Purcell 2006) |
| B2 | Distal enclosure distal flap transition + HEF narrowing segment length | 48 | (Purcell and Almeida 2003 , Rapicavoli et al. 2015) |
| B3 | Distal enclosure distal flap transition triangle base | 11.5 | (Purcell and Almeida 2003 , Newman et al. 2004) |
| B4 | Distal enclosure widening segment (proximal flap transition + medial segment) length | 35 | (Purcell and Almeida 2003 , Newman et al. 2004) |
| B5 | Distal enclosure valve contact patch distal lateral diameter | 14.5 | (Purcell and Almeida 2003 , Newman et al. 2004 , Almeida and Purcell 2006) |
| B6 | Distal enclosure valve contact patch distal depth | 10 | (Newman et al. 2004 , Almeida and Purcell 2006) |
| B7 | Hgl distal enclosure valve contact patch length | 9 | (Purcell and Almeida 2003 , Newman et al. 2004 , Almeida and Purcell 2006) |
| B8 | Egl distal basin valve extension inner medial diameter | 9 | (Almeida and Purcell 2006) |
| B9 | Protruding valve segment length | 4 | (Newman et al. 2004 , Almeida and Purcell 2006 , Rapicavoli et al. 2015) |
| B10 | Protruding valve segment width | 10 | (Newman et al. 2004 , Almeida and Purcell 2006 , Rapicavoli et al. 2015) |
| C1 | Hgl basin distal dip length | 13 | (Newman et al. 2004) |
| C2 | Hgl basin medial hill length | 13 | (Newman et al. 2004) |
| C3 | Egl basin width | 17.5 | (Newman et al. 2004 , Rapicavoli et al. 2015) |
| C4 | Egl fold diameter (parallel to C3) | 0.5*egl basin width | (Newman et al. 2004 , Rapicavoli et al. 2015) |
| C5 | Egl basin cone distal diameter | 17 | (Newman et al. 2004) |
| C6 | Egl basin cone length | 9 | (Newman et al. 2004) |
| C7 | Egl groove at the proximal end of the basin: basin entrance length | 8 | (Newman et al. 2004) |
| C8 | Hgl trough distal diameter | 20 | (Purcell and Almeida 2003 , Newman et al. 2004) |
| C9 | Hgl proximal dip length | 20 | (Newman et al. 2004) |
| C10 | Hgl distal trough length | 38 | (Purcell and Almeida 2003 , Newman et al. 2004) |
| C11 | Egl distal trough distal diameter | 14 | (Newman et al. 2004) |
| C12 | Egl trough length ratio medial:distal | 34:23 | (Newman et al. 2004) |
| C13 | Egl medial-distal trough diameter | 12 | (Newman et al. 2004 , Rapicavoli et al. 2015) |
| C14 | Hgl medial-distal trough diameter | 15 | (Purcell and Almeida 2003 , Newman et al. 2004) |
| C15 | Hgl medial trough length | 42.5 | (Purcell and Almeida 2003 , Newman et al. 2004) |
| C16 | Hgl trough proximal diameter | 20 | (Purcell et al. 1979 , Purcell and Almeida 2003 , Newman et al. 2004 , Rapicavoli et al. 2015) |
| C17 | Egl cibarial-medial trough diameter | 20 | (Purcell et al. 1979 , Newman et al. 2004 , Rapicavoli et al. 2015) |
| C18 | Cibarial trough proximal depth | 67 | (Purcell et al. 1979) |
| C19 | Cibarial trough deep proximal lateral diameter | 9 | (Purcell et al. 1979) |

# Comparison of 3D Model with Micro-CT Measurements

After the 3D model was made using microscopy images, micro-CT measurements of blue-green sharpshooters were published (Ranieri et al. 2020). The micro-CT measurements show a different cibarium shape than our 3D model (Fig. S1).


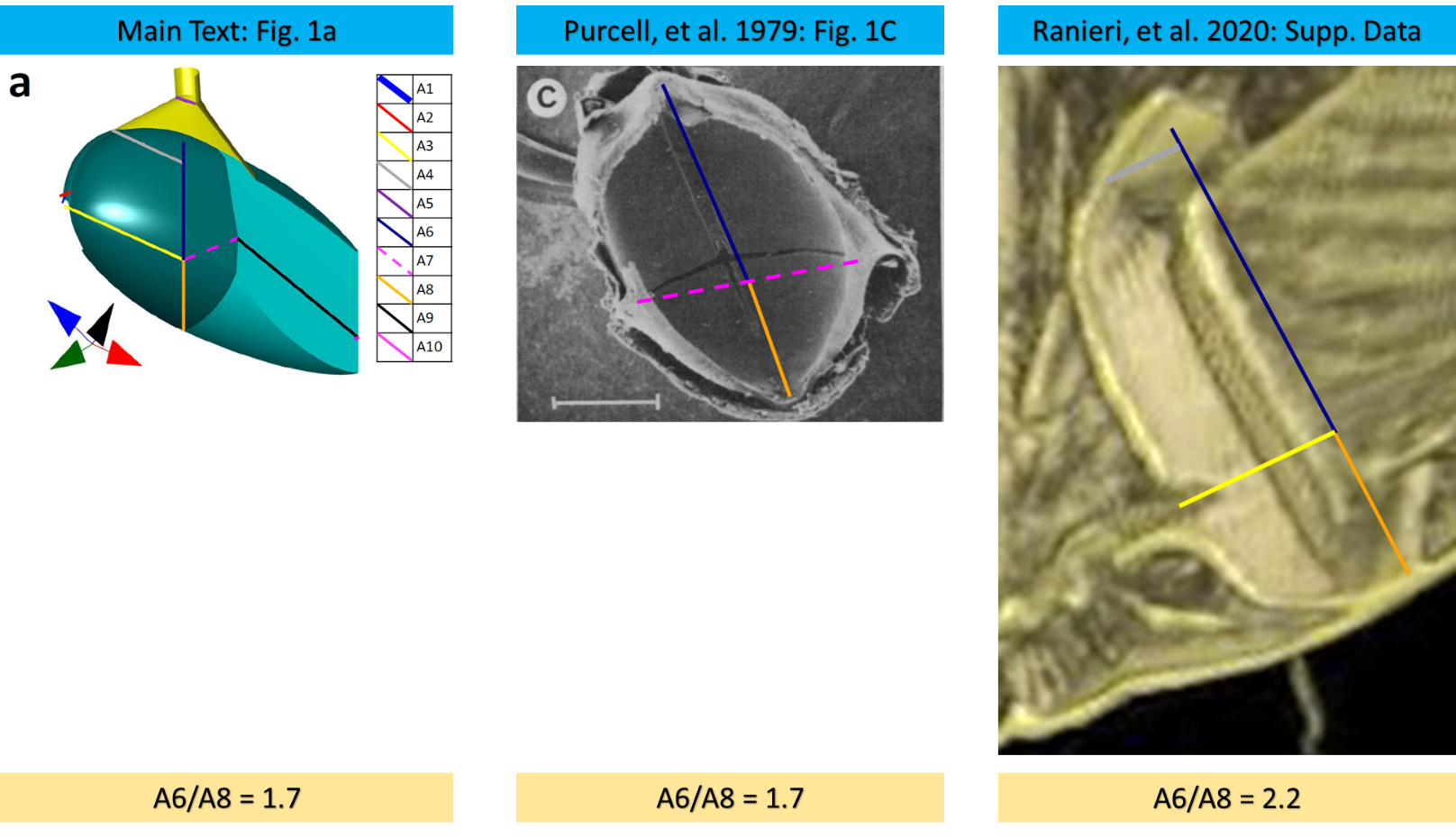


Fig. S1: Comparison of cibarium shape in a microscope image and a micro-CT measurement. The dimensions A6 and A8 in Fig. 1 of the main text are based on measurements from a microscope image published by Purcell, et al. (1979) (From [Purcell, et al. 1979](http://dx.doi.org/10.1126/science.206.4420.839). Reprinted with permission from AAAS.). The ratio of A6 to A8 is different in the image from Purcell, et al. (1979) than in the micro-CT measurement from Ranieri, et al (2020) (Reprinted from Journal of Insect Physiology, Vol 120, Emanuele Ranieri, Gianluca Zitti, Paola Rioloa, Nunzio Isidoro, Sara Ruschioni, Maurizio Brocchini, Rodrigo P.P. Almeida, Fluid dynamics in the functional foregut of xylem-sap feeding insects: A comparative study of two *Xylella fastidiosa* vectors, 103995, Copyright 2020, with permission from Elsevier.). This may be due to biological variability.

The micro-CT study reports a 130° bend in the precibarium (Ranieri et al. 2020). Our 3D model has a 117° bend at that location (Fig. S1), which is within 10% of 130°. The length the of precibarium was reported to be 154 µm based on micro-CT (Ranieri et al. 2020). Our 3D model is 168 µm long between the points where the trough meets the cibarium and where the distal enclosure meets the HEF (Fig. S1). While 168 µm is an underestimate due to the curviness of the precibarium, it is within 10% of 154 µm. Therefore, our 3D model is in reasonable agreement with micro-CT measurements.

Fig. S2: Measurements of the 3D model of the precibarium for comparison with Ranieri et al. (2020).


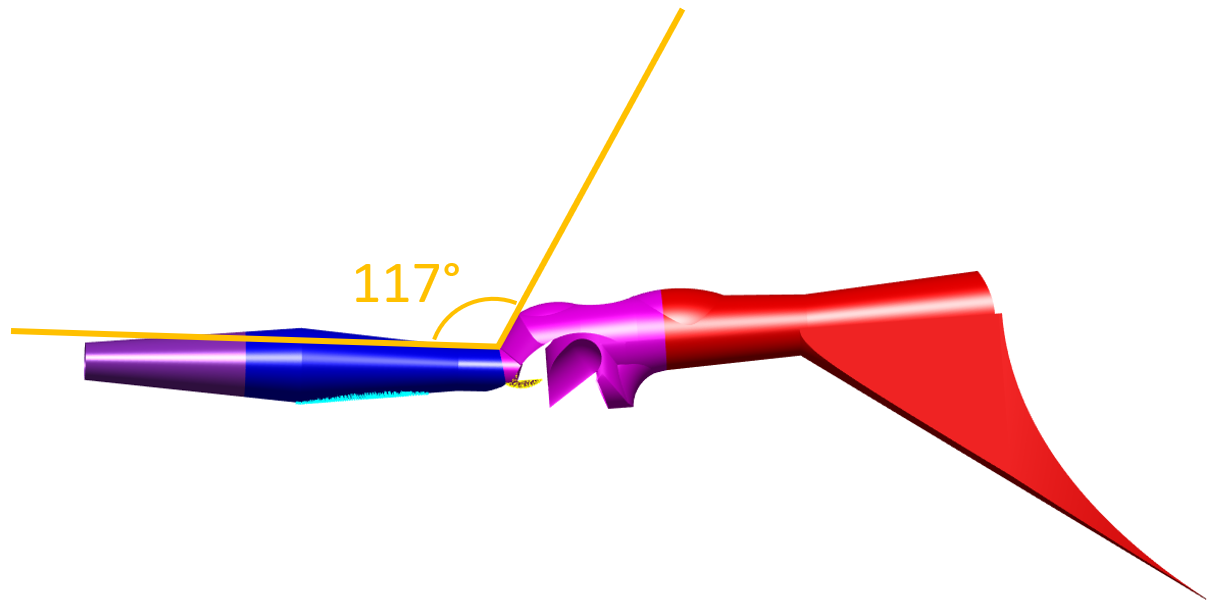


168 µm

# References Cited

**Almeida, R. P., and A. H. Purcell. 2006.** Patterns of *Xylella fastidiosa* colonization on the precibarium of sharpshooter vectors relative to transmission to plants. Annals of the Entomological Society of America 99: 884-890.

**Alves, E., B. Leite, R. C. Marucci, S. F. Pascholati, J. R. Lopes, and P. C. Andersen. 2008.** Retention sites for *Xylella fastidiosa* in four sharpshooter vectors (Hemiptera: Cicadellidae) analyzed by scanning electron microscopy. Current Microbiology 56: 531-538.

**Newman, K. L., R. P. Almeida, A. H. Purcell, and S. E. Lindow. 2004.** Cell-cell signaling controls *Xylella fastidiosa* interactions with both insects and plants. Proceedings of the National Academy of Sciences 101: 1737-1742.

**Purcell, A. H., and R. P. Almeida. Year.** Published. Characterization and studies on the fundamental mechanisms of *Xylella fastidiosa* transmission to grapevines by the glassy-winged sharpshooter, pp. 115 -117. *In*, Pierce’s Disease Research Symposium, 2003.

**Purcell, A. H., A. H. Finlay, and D. L. McLean. 1979.** Pierce's disease bacterium: mechanism of transmission by leafhopper vectors. Science 206: 839-841.

**Ranieri, E., G. Zitti, P. Riolo, N. Isidoro, S. Ruschioni, M. Brocchini, and R. P. Almeida. 2020.** Fluid dynamics in the functional foregut of xylem-sap feeding insects: a comparative study of two *Xylella fastidiosa* vectors. Journal of Insect Physiology 120: 103995.

**Rapicavoli, J. N., N. Kinsinger, T. M. Perring, E. A. Backus, H. J. Shugart, S. Walker, and M. C. Roper. 2015.** O antigen modulates insect vector acquisition of the bacterial plant pathogen *Xylella fastidiosa*. Appl. Environ. Microbiol. 81: 8145-8154.
